# Chemerin in the Spotlight: Revealing Its Multifaceted Role in Acute Myocardial Infarction

**DOI:** 10.3390/biomedicines12092133

**Published:** 2024-09-20

**Authors:** Andreas Mitsis, Elina Khattab, Michael Myrianthefs, Stergios Tzikas, Nikolaos P. E. Kadoglou, Nikolaos Fragakis, Antonios Ziakas, George Kassimis

**Affiliations:** 1 Cardiology Department, Nicosia General Hospital, State Health Services Organization, Nicosia 2029, Cyprus; khattab_elina@outlook.com (E.K.); myr.michael@shso.org.cy (M.M.); 2 Third Department of Cardiology, Aristotle University of Thessaloniki, 54636 Thessaloniki, Greece; tzikas@gmail.com; 3 Medical School, University of Cyprus, Nicosia 2115, Cyprus; kadoglou.nikolaos@ucy.ac.cy; 4 Second Department of Cardiology, Aristotle University of Thessaloniki, 54642 Thessaloniki, Greece; fragakis.nikos@googlemail.com (N.F.); gksup@yahoo.gr (G.K.); 5 First Department of Cardiology, AHEPA University Hospital, Aristotle University of Thessaloniki, 54636 Thessaloniki, Greece; aziakas@auth.gr

**Keywords:** acute myocardial infarction, adipokines, atherosclerosis, biomarkers, cardiovascular disease, cardiovascular risk factors, chemerin, coronary artery disease, inflammation, pro-inflammatory cytokines

## Abstract

Chemerin, an adipokine known for its role in adipogenesis and inflammation, has emerged as a significant biomarker in cardiovascular diseases, including acute myocardial infarction (AMI). Recent studies have highlighted chemerin’s involvement in the pathophysiological processes of coronary artery disease (CAD), where it modulates inflammatory responses, endothelial function, and vascular remodelling. Elevated levels of chemerin have been associated with adverse cardiovascular outcomes, including increased myocardial injury, left ventricular dysfunction, and heightened inflammatory states post-AMI. This manuscript aims to provide a comprehensive review of the current understanding of chemerin’s role in AMI, detailing its molecular mechanisms, clinical implications, and potential as a biomarker for diagnosis and prognosis. Additionally, we explore the therapeutic prospects of targeting chemerin pathways to mitigate myocardial damage and improve clinical outcomes in AMI patients. By synthesizing the latest research findings, this review seeks to elucidate the multifaceted role of chemerin in AMI and its promise as a target for innovative therapeutic strategies.

## 1. Introduction

Acute myocardial infarction (AMI) remains a leading cause of morbidity and mortality worldwide [1]. Despite advancements in medical therapies and interventional strategies, the prognosis of AMI patients can be markedly influenced by the extent of myocardial damage and the subsequent inflammatory response [2]. Therefore, identifying novel biomarkers and therapeutic targets to better understand and manage AMI is of paramount importance [3].

Chemerin, an adipokine primarily known for its roles in adipogenesis and inflammation, has recently earned attention in the field of cardiovascular research. Originally identified as a chemoattractant protein, chemerin is involved in various physiological processes, including immune cell migration and vascular biology [4]. Elevated chemerin levels have been implicated in several metabolic and inflammatory conditions, highlighting its potential significance in cardiovascular diseases [5].

This manuscript aims to provide a comprehensive review of the emerging role of chemerin in the context of AMI. After analyzing the pivotal role of adipose tissue in cardiovascular disease (CVD) pathophysiology, we will focus on the molecular mechanisms through which chemerin influences the pathophysiology of atherosclerosis, its impact on inflammatory responses, endothelial function, and vascular remodelling. Furthermore, we will explore the clinical implications of chemerin as a biomarker for diagnosing and prognosticating AMI, as well as its potential as a therapeutic target. By synthesizing the latest research findings, this review seeks to elucidate the multifaceted role of chemerin in AMI and its promise as a target for innovative therapeutic strategies.

## 2. Adipose Tissue: A Central Player in Cardiovascular Disease Pathogenesis

Adipose tissue, traditionally viewed as a passive storage site for excess energy, is now recognized as a highly active endocrine organ that plays a significant role in CVDs.

Adipose tissue secretes a wide range of bioactive molecules known as adipokines. Adipokines play pivotal roles in regulating inflammation and metabolic processes. While certain adipokines, such as adiponectin and resistin, are often considered as hormones due to their endocrine functions [6], others, including chemerin, modulate inflammatory responses and vascular function, contributing to the pathophysiology of cardiovascular diseases like acute myocardial infarction [7,8]. These adipokines have profound effects on systemic inflammation, glucose metabolism, and lipid homeostasis, all of which are critical in the pathogenesis of CVDs [9].

Some adipokines, including TNF-α, IL-6, visfatin, and leptin, are recognized as pro-inflammatory cytokines that promote the progression of atherosclerosis and contribute to the development of acute coronary syndromes (ACSs) [3,10]. Conversely, other adipokines like adiponectin are anti-inflammatory, playing a protective role against atherosclerosis [11,12]. The relationship between the blood levels of these anti-inflammatory molecules and ACS has been widely studied [13].

Excess adipose tissue, particularly in the visceral region, contributes to a chronic low-grade inflammatory state [14]. This inflammation is mediated through the increased secretion of pro-inflammatory adipokines and decreased levels of protective ones like adiponectin. The inflammation from adipose tissue can lead to endothelial dysfunction, characterized by impaired nitric oxide (NO) production and increased vascular stiffness, which are early markers of atherosclerosis [15]. Furthermore, this inflammatory background promotes the development of insulin resistance, dyslipidemia and hypertension—key components of the metabolic syndrome, which is a major risk factor for CVDs [16].

Visceral adiposity also contributes to the accumulation of ectopic fat in organs like the liver, heart, and muscles, which exacerbates metabolic disturbances and further increases cardiovascular risk [17,18]. The interplay between adipose tissue and other metabolic organs, such as the liver and pancreas, is also crucial [19]. Excess free fatty acids released from adipose tissue can lead to fatty liver disease and pancreatic beta-cell dysfunction, both of which are associated with an increased risk of cardiovascular events [20]. Finally, obesity-related hypoxia in adipose tissue can lead to adipocyte death and macrophage infiltration, which amplifies the inflammatory response and accelerates the development of atherosclerotic plaques [21]. This plaque formation, if unstable, can lead to acute coronary events like myocardial infarction.

## 3. Chemerin: Molecular and Biological Insights

Chemerin is a 16–18 kDa adipokine primarily found in white adipocytes, where it functions as a leukocyte chemoattractant [22]. Identified in 2003 as the ligand for the G protein-coupled receptor (GPCR) chemerin-like receptor 1 (CMKLR1, also known as ChemR23), chemerin is produced by the retinoic acid receptor responder protein 2 (RARRES2) gene, which encodes a 163-amino-acid protein [23]. The most active form of chemerin, chemerin21–157, is generated through C-terminal processing by serine proteases of the 143-amino-acid precursor protein, pro-chemerin [24]. The C-terminus is vital for chemerin’s biological activity, as modifications in this region significantly affect its potency [25]. In addition to activating CMKLR1, human chemerin21–157, chemerin-9, and chemerin-13 also activate the orphan receptor G protein-coupled receptor 1 (GPR1) due to its high sequence similarity with CMKLR1 [26]. Chemerin interacts with several receptors, including CMKLR1, GPR1, and chemokine C-C motif receptor-like 2 (CCRL2) [27]. While CMKLR1 is widely expressed in human tissues such as dendritic cells, macrophages, monocytes, adipose tissue, and endothelial cells, GPR1 has a broader expression in adipose tissue, the brain, and various reproductive tissues [28,29,30,31]. CCRL2, expressed in neutrophils, macrophages, dendritic cells, and T lymphocytes, acts as a chaperone protein, localizing chemerin to enhance its interaction with CMKLR1 [32]. Unlike CMKLR1 and GPR1, CCRL2 does not activate downstream signalling, classifying it as an atypical chemokine receptor [33].

Chemerin’s role in inflammation and atherosclerosis is multifaceted. It activates key signalling pathways such as mitogen-activated protein kinase (MAPK) and nuclear factor-kappa B (NF-κB), which regulate endothelial cell functions like migration, proliferation, and angiogenesis [5]. Additionally, chemerin plays a critical role in directing immune cell migration to inflamed tissues, contributing to vascular inflammation [34]. It can activate pro-inflammatory cytokines, such as TNF-α and interleukin-1b (IL-1β), facilitating the recruitment and activation of macrophages, dendritic cells, and other antigen-presenting cells through the CMKLR1 receptor, thereby accelerating the progression of atherosclerosis [29,35,36]. Conversely, chemerin can also act as an anti-inflammatory mediator by inhibiting neutrophil and TNF-α-induced vascular cell adhesion molecule-1 (VCAM-1) expression, monocyte recruitment, and reducing cytokine and chemokine production in various inflammatory models [37]. This suggests a complex, context-dependent role in inflammation. Clinical studies have shown that chemerin levels correlate positively with inflammatory markers such as high-sensitivity C-reactive protein (hs-CRP), IL-6, TNF-α, and metabolic markers like leptin, triglycerides, low-density lipoprotein (LDL), and apolipoprotein B, as well as body mass index (BMI) [38,39,40,41]. Chemerin’s production is stimulated by TNF-α, and it is expressed in dendritic cells and macrophages, where it attracts monocytes and macrophages [42].

Chemerin’s involvement in atherosclerosis is further evidenced by its ability to enhance leukocyte adhesion and migration via β2 integrins and extracellular signal-regulated kinase 1/2 (ERK1/2) signalling [43]. Recent experimental studies have shown that chemerin, regulated by CCRL2, enhances leukocyte adhesion and migration by activating these integrins and promoting ERK1/2 signalling, playing a crucial role in the progression of atherosclerosis [44]. Another study involving ApoE-/- mice demonstrated that chemerin enhances the adhesion and migration abilities of endothelial progenitor cells, increasing plaque instability and abnormal lipid accumulation through the p38 MAPK pathway [45]. Furthermore, Yanofsky et al. revealed a positive correlation between carotid plaque instability and increased protein levels of chemerin and ChemR23, but a negative association with chemerin mRNA expression, suggesting a pro-inflammatory role in atherosclerosis through a negative feedback regulatory mechanism [46].

Chemerin acts as a chemoattractant protein, intensifying immune cell adhesion at inflammation sites [47,48]. It enhances the expression of endothelial adhesion molecules, such as E-selectin and VCAM-1, and promotes monocyte–endothelial adhesion via the NF-κB, MAPK, and phosphatidylinositol 3-kinase/Akt (PI3K/Akt) pathways [49]. It acts as a vascular endothelial growth factor, significantly promoting blood vessel formation, vascular smooth muscle cell (VSMC) proliferation, carotid intimal hyperplasia, and vascular remodelling through the PI3K/Akt and MAPK pathways [49]. Additionally, chemerin interacts with receptors CMKLR1 and CCRL2, affecting endothelial cells, smooth muscle cells, and macrophages [50]. Elevated chemerin levels are linked to metabolic syndrome [51], insulin resistance [52], and lipid metabolic dysfunction by impacting signalling pathways like ERK [53], underscoring its role as a potential risk factor for atherosclerosis and metabolic imbalance [54]. Similarly, Zhang et al. showed that chemerin can induce insulin resistance in rat cardiomyocytes, in part through the ERK1/2 pathway [55]. Of note, research has shown that in the presence of obesity, adipocyte-derived chemerin might function as an endogenous cardioprotective factor against obesity-related cardiomyopathy [56].

**Figure 1:**
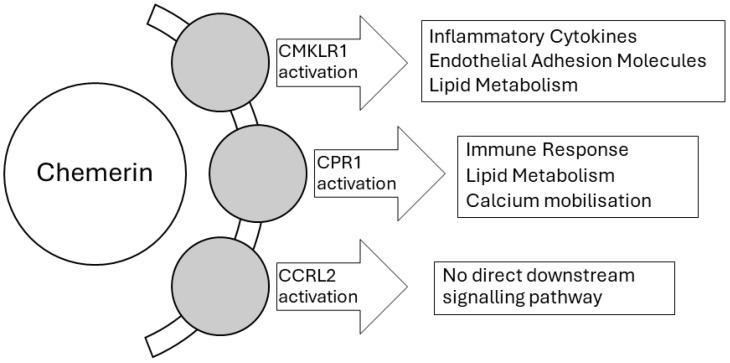
Chemerin Signalling Pathways: Molecular Interactions in Inflammation, Endothelial Dysfunction, and Lipid Metabolism.

Finally, chemerin modulates vascular endothelial function under stress by enhancing the expression of endothelial cell adhesion molecules and initiating inflammatory responses [49,57], while it has been found to regulate autophagy and mitochondrial reactive oxygen species (ROS) production [58]. This extensive distribution of chemerin receptors in various organs indicates that chemerin influences a wide range of tissues and cells, including endothelial and inflammatory cells (Figure 1).

This diagram illustrates the molecular pathways activated by chemerin, highlighting its interaction with CMKLR1 (ChemR23) receptor and the downstream effects on various biological processes. Upon activation of CMKLR1, chemerin influences several key mechanisms including:

**Inflammatory Cytokine Production**: Chemerin promotes the secretion of pro-inflammatory cytokines such as TNF-α and IL-6, contributing to enhanced inflammation, particularly in the context of cardiovascular diseases like acute myocardial infarction (AMI).

**Endothelial Adhesion Molecule Expression**: Chemerin upregulates the expression of endothelial adhesion molecules (e.g., VCAM-1, ICAM-1), facilitating leukocyte adhesion and endothelial dysfunction, which are pivotal in vascular inflammation and remodelling.

**Lipid Metabolism**: Chemerin plays a role in lipid metabolism regulation, influencing processes such as lipid accumulation and plaque instability, thus contributing to the progression of atherosclerosis and related cardiovascular diseases.

CCRL2 does not have a direct downstream signalling pathway. Unlike CMKLR1 and GPR1, which initiate intracellular signalling when activated by chemerin, CCRL2 functions as a “decoy” or “atypical” receptor. Its primary role is to bind chemerin and present it to other receptors, like CMKLR1, without triggering intracellular signalling itself.

## 4. Chemerin in Cardiovascular Diseases

Chemerin is closely linked to cardiovascular health (Table 1), particularly in the context of inflammation, endothelial dysfunction, metabolic disorders, angiogenesis, and calcification [15,32,59,60]. Adipogenesis, endothelial dysfunction, and inflammation are key features in the progression of coronary artery disease (CAD), significantly contributing to poor prognosis and increased CVD mortality [22,61]. Chemerin concentrations may serve as an independent biomarker for arterial integrity and the early stages of atherosclerosis, as well as a novel therapeutic target [62,63]. As a growth factor, chemerin induces metalloproteases such as matrix metalloproteinase-7 (MMP-7), MMP-2, and MMP-9, which promote endothelial angiogenesis and influence blood vessel growth and remodelling [64]. As a chemokine, chemerin attracts immune cells like natural killer cells, macrophages, and dendritic cells, modulating endothelial adhesion molecule levels [65]. It induces the expression of E-selectin and intercellular adhesion molecule-1 (ICAM-1) in coronary artery endothelial cells and is synthesized in perivascular adipose tissue [66]. Moreover, chemerin activates ChemR23 in VSMC, promoting contraction and a pro-inflammatory phenotype [67]. It also regulates NO signalling in endothelial cells, highlighting its role as an endogenous vasoregulator [68], and as an independent risk factor for arterial stiffness [69].

Elevated levels of chemerin have been associated with adverse cardiovascular events in CAD and heart failure patients, indicating its potential as a novel risk factor for CVD development [70,71,72,73]. Chemerin is a versatile protein that has recently been recognized as a crucial factor in conditions such as hypertension, myocardial infarction, CAD, preterm birth, diabetes, and metabolic diseases [74,75,76,77,78,79]. The levels of chemerin and its receptor CMKLR1 in periaortic and peri coronary fat [80] and foam cells are linked to the severity of atherosclerosis and the instability of carotid plaques [46]. Furthermore, chemerin has been implicated in hypertension arterial contraction and the sensitivity of the sympathetic nervous system [81] or by the endogenous modification of the sympathetic nerve-mediated contraction through ChemR23 and that ChemR23 receptors [82]. In pulmonary arterial hypertension (PAH), chemerin-9, a ChemR23 agonist, promotes intrapulmonary artery contraction by upregulating ChemR23 expression in VSMC [83].

Elevated concentrations of circulating chemerin are related to the presence of CAD [84,85,86]. Interestingly, a recent meta-analysis of five studies involving patients with ACS demonstrated that chemerin levels were significantly elevated in ACS patients compared to controls [87]. The analysis also showed that plasma chemerin levels were even higher in diabetic patients, reinforcing the association between chemerin and both cardiovascular and metabolic conditions.

Another study involving 256 patients with atrial fibrillation (AF) found that serum concentrations of chemerin were elevated in AF patients, particularly in those with persistent AF, compared to those with paroxysmal or persistent AF [88]. This study suggests that chemerin may contribute to AF development through its role in inflammation and atrial remodelling, with serum levels correlating with metabolic parameters and left atrial diameter, which is a marker of atrial remodelling. Chemerin has also been linked to vascular calcification [60]. Chemerin-9 appears to inhibit phosphate-induced calcification in VSMCs by increasing the expression of matrix Gla protein, a calcification inhibitor; however, this effect is not observed in cells lacking ChemR23, indicating a complex regulatory role in vascular calcification [89]. Interestingly, Lurins et al. demonstrated that chemerin levels are elevated in patients with aortic valve stenosis, indicating a potential role for chemerin in calcification and the inflammatory processes associated with the condition [90]. Finally, chemerin’s involvement in diabetic cardiomyopathy has been demonstrated through its role in the chemerin/CMKLR1 axis, which contributes to inflammation, hypertrophy, proptosis, and fibrosis in the heart tissue of DBCM rats, primarily via NLRP3 inflammasome activation [91].

## 5. Chemerin: A Key Pathway in the Pathophysiology of Acute Myocardial Infarction

Chemerin has been proposed to play a multifaceted role in the pathophysiology of AMI. It can act as a chemokine, attracting other cytokines through the vasculature [29]; as an adipokine adjusting lipid and glucose levels [32,92], potentially affecting their infiltration into the endothelium; and as a growth factor affecting angiogenesis [93]. It exerts its effects primarily through its interaction with the chemerin receptors, particularly CMKLR1, which is expressed on various cell types including endothelial cells, smooth muscle cells, mast cells, and immune cells such as macrophages [94]. During AMI, chemerin is upregulated and contributes to the recruitment of immune cells to the site of injury, amplifying the inflammatory response [95]. This heightened inflammatory activity is crucial in the initial stages of myocardial infarction, aiding in the clearance of necrotic cells and initiating tissue repair [96]. However, excessive chemerin signalling can exacerbate inflammation [97], leading to adverse cardiac remodelling, increased infarct size, and progression to heart failure. Chemerin also influences lipid metabolism [98] and may contribute to the instability of atherosclerotic plaques, thereby increasing the risk of plaque rupture and subsequent myocardial infarction [99].

Chemerin significantly modulates inflammatory responses during AMI by acting as a chemotactic agent that recruits and activates various immune cells, including macrophages and dendritic cells, to the ischemic myocardium [29,100]. This recruitment is mediated by the chemerin receptor CMKLR1, which, upon binding to chemerin, triggers a cascade of intracellular signalling pathways that promote the production of pro-inflammatory cytokines such as IL-6 and TNF-α [49,101]. These cytokines further amplify the inflammatory environment within the infarcted myocardium, contributing to the breakdown of extracellular matrix and facilitating the infiltration of additional inflammatory cells [102,103,104]. Interestingly, CMKLR1 deficiency delays macrophage phenotypic transformation and cardiac repair following MI through the PI3K/AKT/mTOR pathway. Therefore, CMKLR1 could serve as a promising therapeutic target for MI [105]. Chemerin’s role in balancing these inflammatory processes highlights its potential as a therapeutic target to modulate the inflammatory response in AMI, aiming to minimize tissue damage while supporting necessary repair mechanisms.

Chemerin also plays a critical role in endothelial function and vascular remodelling during AMI. It affects endothelial cells by promoting the expression of adhesion molecules such as VCAM-1 and ICAM-1 [49,106], which facilitate the adhesion and transmigration of leukocytes into the vascular wall [107]. This process not only contributes to the inflammatory response, but also to the destabilization of the endothelium, which can exacerbate ischemic injury [108]. This might be facilitated via ROS accumulation in endothelial cells, contributing to endothelial dysfunction [109]. Furthermore, chemerin influences vascular smooth muscle cells, promoting their migration and proliferation, which are key processes in vascular remodelling [110,111,112]. This remodelling can lead to pathological changes in the coronary arteries, such as intimal hyperplasia and increased arterial stiffness, which impair blood flow and exacerbate myocardial ischemia [113]. Additionally, chemerin-induced endothelial dysfunction is linked to NO bioavailability, which further compromises vascular reactivity and increases the risk of thrombus formation [114,115]. The combined effects of chemerin on endothelial cells and vascular smooth muscle cells underscore its significant impact on the structural and functional changes in the vasculature following AMI, making it a potential target for therapies aimed at preserving endothelial function and preventing adverse vascular remodelling.

## 6. Clinical Implications of Chemerin in Acute Myocardial Infarction

As research continues to uncover the complex mechanisms underlying AMI, the role of novel biomarkers has become increasingly significant in both diagnosis and prognosis. Among these, chemerin has the potential not only as a marker of inflammation, but also as a valuable tool for assessing disease severity and predicting patient outcomes [116].

Chemerin levels have been found to correlate with various cardiovascular outcomes in patients experiencing AMI. During an AMI, chemerin levels rise in conjunction with acute inflammatory markers such as CRP, suggesting its role as an early indicator of myocardial injury [117]. This correlation highlights chemerin’s utility in the early diagnosis of AMI, where timely detection is critical for initiating appropriate therapeutic interventions. Interestingly, the plasma chemerin concentration is elevated in patients with ACS but not in these with stable angina pectoris [118,119]. Furthermore, elevated plasma chemerin concentrations are associated with more severe forms of AMI, larger infarct sizes, and a worse overall prognosis rather than cases of unstable angina [118,120]. Elevated chemerin levels have been found in ST-elevation MI patients with a high thrombotic burden [117]. Moreover, studies have shown that higher chemerin levels are linked to increased inflammation, adverse cardiac remodelling, and a higher incidence of complications such as heart failure and recurrent myocardial infarctions [121]. This correlation suggests that chemerin not only reflects the extent of myocardial injury, but also plays a role in the progression of disease and the long-term cardiovascular outcomes of AMI patients [71,119]. Chemerin’s association with the severity of infarction and adverse outcomes suggests it could be a valuable prognostic marker. This predictive capability makes chemerin particularly useful in assessing the risk of complications following an AMI, thus aiding in patient risk stratification.

Another clinical application of chemerin could be the assessment of plaque stability in the context of AMI. Just as elevated chemerin levels have been linked to unstable atherosclerotic plaques in the carotid arteries [46], it is likely that similar elevations would be observed in unstable coronary plaques, which are prone to rupture, leading to the onset of AMI. Chemerin contributes to plaque instability by promoting inflammatory processes within the plaque, including the recruitment and activation of macrophages through the CMKLR1 receptor [46]. This heightened inflammatory state can weaken the fibrous cap of the plaque, making it more susceptible to rupture.

Studies have demonstrated that patients with higher chemerin levels exhibit a greater likelihood of having unstable plaques, thus increasing the risk of AMI and other adverse cardiovascular events [70]. This makes chemerin not only a biomarker for detecting AMI, but also a crucial tool in predicting the risk of plaque rupture. By integrating chemerin measurement into clinical practise, healthcare providers could better identify patients at higher risk of plaque instability, allowing for more proactive management strategies aimed at preventing plaque rupture and subsequent myocardial infarction. This highlights the potential of chemerin as a valuable prognostic biomarker in cardiovascular disease management.

Given its involvement in inflammatory response and vascular remodelling, chemerin holds significant potential as a biomarker for both the diagnosis and prognosis of AMI. As chemerin levels rise in parallel with acute inflammatory markers like CRP during an AMI, it could serve as an early indicator of myocardial injury. Additionally, chemerin’s ability to predict the severity of the infarction and its association with poor outcomes make it a promising prognostic marker. The integration of chemerin measurement into clinical practise could enhance the accuracy of AMI diagnosis and provide valuable insights into patient risk stratification, guiding more personalized therapeutic interventions and improving long-term outcomes (Table 2).

## 7. Therapeutic Prospects of Targeting Chemerin in Cardiovascular Diseases: Current Status

Chemerin has emerged as a potential therapeutic target in cardiovascular diseases. Therapeutic strategies targeting chemerin pathways are still in the early stages, mostly in pre-clinical development, with research primarily concentrated on understanding its role in inflammation, endothelial function, and lipid metabolism [122]. The most attention has been paid to the significant role of the chemerin/chemR23 axis [57]. This signalling pathway is not only pivotal in modulating inflammatory responses, but also plays a significant role in vascular remodelling and plaque stability, making it a key focus in the study of CVDs. The chemerin/ChemR23 axis, through its interactions with various cell types, including macrophages and vascular smooth muscle cells, can influence both the progression of atherosclerosis and the severity of myocardial injury during AMI.

CCX832 is a small-molecule antagonist of the chemerin/chemR23 axis and works by specifically binding to ChemR23, thereby blocking the interaction of chemerin with its receptor [123]. This inhibition prevents the downstream signalling that normally occurs when chemerin activates ChemR23, leading to reduced inflammation and other associated pathological processes [123,124]. CCX832 enhanced chemerin-induced vascular inflammation in human microvascular ECs and human aorta endothelial cells [125]. Neves et al. found that the chemerin/chemR23 axis plays a critical role in diabetes-associated vascular oxidative stress and altered insulin signalling. Targeting chemerin/chemR23 may be an attractive strategy to improve insulin signalling and vascular function in obesity-associated diabetes [68].

Another agent that modifies the chemerin/ChemR23 axis is resolvin E1 (RvE1) [126]. RvE1 is a specialized pro-resolving lipid mediator derived from the omega-3 fatty acid eicosapentaenoic acid. RvE1 is part of the resolvin family, which plays a crucial role in actively resolving inflammation and returning tissues to homeostasis after an inflammatory response [127]. RvE1 binds to ChemR23 and inhibits the recruitment and activation of inflammatory cells like neutrophils and macrophages, reducing the production of pro-inflammatory cytokines and chemokines, reducing chemerin-mediated vascular dysfunction [128].

Among the various synthetic agonists of chemerin that activate downstream signalling at CMKLR1 and GPR1 [129], chemerin-9 (C9) appears to be the more potent [123]. C9 represents the nine C-terminal amino acids of chemerin, which are crucial for binding to the chemerin receptor, ChemR23, mimicking the natural ligand chemerin but in a more controlled manner. Chen et al. found that C9 treatment significantly restrained inflammatory cell infiltration, neovascularization, and MMP expression, while increasing the elastic fibres and smooth muscle cells in Ang II-induced abdominal aortic aneurysm in ApoE^−/−^ mice [130]. Similarly, Sato et al. showed that C9 significantly suppressed the TNF-α-induced adhesion of pro-inflammatory monocytes to smooth cells and macrophage inflammatory phenotypes, and markedly diminished the areas of aortic atherosclerotic lesions [131]. These approaches aim to reduce chemerin’s pro-inflammatory effects and its role in vascular remodelling, which are critical in the context of ACS.

Finally, antisense oligonucleotides (ASOs) are being explored for their potential to modulate chemerin’s effect. ASOs are short, synthetic strands of nucleic acids designed to bind specifically to the mRNA of a target gene. By binding to the mRNA, ASOs can inhibit the expression of the corresponding gene, effectively “silencing” it [132]. This is achieved through various mechanisms, including the degradation of the mRNA by RNase H, blocking the translation of the mRNA into protein, or altering mRNA splicing [133]. Ferland et al. demonstrated that reducing chemerin levels through ASOs significantly lowers blood pressure in high-fat diet-induced hypertension in rats, with a greater effect observed compared to high-salt diet-induced hypertension [134].

## 8. Therapeutic Prospects of Targeting Chemerin in Acute Myocardial Infarction: Challenges and Future Directions

The potential benefits of chemerin-targeted therapies in AMI are many. First, by reducing chemerin’s pro-inflammatory effects, such therapies could mitigate the inflammatory response following myocardial infarction, which is known to contribute to adverse cardiac remodelling and heart failure and remains a major target of anti-inflammatory therapy [135]. Additionally, chemerin-targeted therapies may improve endothelial function and reduce vascular stiffness, thereby enhancing coronary perfusion and reducing the risk of recurrent ischemic events [136]. Another potential benefit is the normalization of lipid metabolism, which could help in stabilizing atherosclerotic plaques and preventing plaque rupture, a common cause of AMI [137].

Despite these potential benefits, there are several challenges associated with the proposed chemerin-targeted therapies. One significant challenge is the dual role of chemerin, as it also has protective effects under certain conditions, such as in the regulation of adipocyte differentiation and metabolism. Therefore, inhibiting chemerin could lead to unintended consequences, such as metabolic dysregulation. Moreover, the complexity of chemerin’s interaction with its receptors (CMKLR1, GPR1, and CCRL2) and the involvement of these receptors in various physiological processes complicate the development of specific and safe chemerin-targeted therapies. Another challenge is the potential for off-target effects, given chemerin’s widespread expression in various tissues beyond the cardiovascular system. Finally, the long-term effects of chemerin inhibition in patients with AMI are not yet fully understood, raising concerns about the possible safety and efficacy of such treatments.

In comparison to other emerging therapeutic targets for ACS and AMI [138], chemerin offers a unique advantage due to its multifaceted role in inflammation, endothelial dysfunction, and lipid metabolism. For example, while IL-6 is primarily involved in modulating the inflammatory response [139], chemerin not only drives inflammation but also directly impacts vascular remodelling and metabolic processes, such as lipid handling and plaque stability. This distinguishes chemerin from other inflammatory markers like CRP, which serves primarily as a systemic marker of inflammation without directly influencing vascular or metabolic pathways [140]. Similarly, when comparing with studies targeting MCP-1, which plays a key role in immune cell recruitment [141], chemerin adds an additional layer of complexity by influencing VSMCs and endothelial function, which are critical in the progression of atherosclerosis and plaque destabilization. Hence, targeting chemerin presents a broader therapeutic opportunity, combining anti-inflammatory effects with vascular protection.

Future therapeutic interventions targeting chemerin in AMI will likely focus on refining the specificity and safety of these approaches. One promising direction is the development of selective antagonists or modulators of chemerin receptors, particularly CMKLR1, which could inhibit the harmful effects of chemerin in AMI, while preserving its beneficial roles in other tissues. Gene therapy or RNA-based approaches might also be explored to downregulate chemerin expression in a tissue-specific manner, reducing the risk of systemic side effects [142]. Another area of interest is the investigation of combination therapies that target multiple pathways involved in AMI, including chemerin, to achieve a more comprehensive therapeutic effect. Additionally, personalized medicine approaches could be employed to identify patients who would benefit most from chemerin-targeted therapies based on their genetic and metabolic profiles [143].

Looking forward, therapeutic strategies targeting chemerin signalling in AMI could focus on selectively modulating its receptor pathways, particularly CMKLR1, to mitigate the adverse inflammatory and vascular effects of chemerin, while preserving its potential protective roles [144]. Small-molecule inhibitors or monoclonal antibodies that block chemerin’s interaction with its receptors could reduce infarct size, promote cardiac repair, and prevent adverse cardiac remodelling post-AMI. RNA-based therapies, such ASOs, also hold promise in the tissue-specific modulation of chemerin expression [145], reducing the risk of systemic side effects. Future clinical trials should evaluate the safety and efficacy of these interventions, particularly in improving AMI outcomes like reduced infarct size, better cardiac function, and the prevention of heart failure. Moreover, integrating chemerin as a biomarker in clinical practise could help personalize treatments by identifying high-risk patients who would benefit the most from chemerin-targeted therapies. Further preclinical and clinical research will be essential to understand the full therapeutic potential and limitations of chemerin-targeted interventions in AMI, paving the way for innovative treatments that improve patient outcomes.

## 9. Conclusions

In summary, chemerin has emerged as a multifaceted player in the pathophysiology of CVDs. Its role extends beyond its mere involvement in inflammatory processes to influencing vascular remodelling, endothelial function, and lipid metabolism—all of which are critical in the progression of atherosclerosis and the onset of an AMI. The elevation of chemerin levels during AMI, in conjunction with other inflammatory markers, underscores its potential as both a diagnostic and prognostic biomarker. This dual role highlights the promise of chemerin as a target for early detection and risk stratification, allowing for more personalized therapeutic interventions.

Furthermore, the therapeutic prospects of targeting the chemerin signalling pathway, particularly through its receptor CMKLR1, offer exciting avenues for future research. These strategies could potentially mitigate the adverse inflammatory responses and vascular dysfunction associated with AMI, ultimately improving patient outcomes. However, the complex, context-dependent nature of chemerin’s effects necessitates further investigation to fully elucidate its therapeutic potential and to ensure the safety and efficacy of chemerin-targeted interventions. As research progresses, the integration of chemerin into clinical practise could revolutionize the management of AMI, enhancing both diagnostic accuracy and treatment efficacy.

## Figures and Tables

**Table 1:** Chemerin’s role in cardiovascular pathophysiology.

Pathophysiological Role	Details	Mechanisms Involved
Inflammatory Response	Chemerin acts as a chemoattractant, recruiting immune cells to sites of injury during AMI, enhancing inflammation.	Activation of CMKLR1 receptor; NF-κB and MAPK pathways.
Endothelial Dysfunction	Chemerin promotes the expression of adhesion molecules, contributing to leukocyte adhesion and endothelial damage.	Increased expression of VCAM-1, ICAM-1, and E-selectin.
Vascular Remodelling	Chemerin influences the proliferation and migration of VSMCs, contributing to vascular remodelling and increased arterial stiffness.	Activation of PI3K/Akt, ERK1/2, and MAPK pathways.
Lipid Metabolism and Plaque Stability	Chemerin is involved in lipid metabolism, which may contribute to atherosclerotic plaque instability and increased risk of AMI.	Regulation of lipid metabolism enzymes; influence on plaque stability.

AMI: acute myocardial infarction; CMKLR1: chemerin-like receptor 1; ERK: extracellular signal-regulated kinase; ICAM-1: intercellular adhesion molecule 1; MAPK: mitogen-activated protein kinase; NF-κB: nuclear factor kappa-light-chain-enhancer of activated B cells; PI3K: phosphatidylinositol 3-kinase; VCAM-1: vascular cell adhesion molecule 1; VSMCs: vascular smooth muscle cells.

**Table 2:** Chemerin as a biomarker in acute myocardial infarction (AMI).

Clinical Application	Chemerin Level	Significance
Diagnosis of AMI	Elevated during AMI in conjunction with other inflammatory markers (e.g., CRP).	Early indicator of myocardial injury, enhancing diagnostic accuracy.
Prognosis of AMI	Higher levels associated with more severe AMI, larger infarct sizes, and worse outcomes.	Predicts the severity of AMI and long-term prognosis, aiding in risk stratification.
Assessment of Plaque Stability	Elevated chemerin levels linked to unstable atherosclerotic plaques.	Helps predict the risk of plaque rupture and subsequent myocardial infarction.
Potential Therapeutic Target	Modulation of chemerin signalling pathways offers potential for therapeutic intervention.	Targeting chemerin pathways could mitigate adverse inflammatory and vascular responses in AMI patients.

AMI: acute myocardial infarction; CRP: C-reactive protein.
